# Functional Outcomes Over 5 Years in First-Episode Schizophrenia Patients: Key Insights from a Longitudinal Cohort Study in Turkey

**DOI:** 10.1192/j.eurpsy.2025.713

**Published:** 2025-08-26

**Authors:** E. Ince Guliyev, S. N. Karabulut, M. Ceylan, C. C. Türkoğlu, C. Horasan, A. Üçok

**Affiliations:** 1 Psychiatry, Istanbul University Faculty of Medicine, Istanbul, Türkiye

## Abstract

**Introduction:**

First-episode schizophrenia (FES) is a critical period where early intervention can influence long-term outcomes. Tracking functional changes and finding their correlates are essential for understanding the disease process.

**Objectives:**

This study aims to evaluate the progression of functional outcomes over a five-year follow-up in FES patients and to examine the clinical correlates of functioning in the fifth year.

**Methods:**

This cohort study included 197 FES patients admitted to the Istanbul Faculty of Medicine Department of Psychiatry Psychotic Disorders Research Program. Global Assessment of Functioning (GAF), Brief Psychiatric Rating Scale(BPRS), and Scale for the Assessment of Negative Symptoms(SANS) scores were recorded, and a comprehensive neuropsychological battery was applied at baseline. FES patients were evaluated regularly with the same clinical scales and GAF scores. The baseline clinical and cognitive parameters and clinical parameters at 1st, 2nd and 5th years were compared with GAF scores over the years. A repeated measures ANOVA was conducted to examine the effect of time on GAF scores over a 5-year follow-up, as well as the effects of gender and education. SPSSv29 was used to conduct all statistical analyses, and significance levels were set at p<0.05.

**Results:**

Seventy-seven FES patients had a follow-up duration of at least five years. Of these, 36.4% (n=28) were female, and the mean age was 22.18±6.09 years. The mean follow-up duration was 133.09±56.94 months. The mean GAF scores were 51.44±12.71 at baseline, 60.00±9.48 at the end of the first year, 62.14±9.04 at the end of the second year, and 62.89±8.34 at the end of the fifth year. There was a significant effect of time on GAF scores, F(2.56, 182.8) = 26.43, p < 0.001, partial η² = 0.29, with scores improving significantly from baseline to year 1 (p<0.001), and further improving by year 5 (p=0.034). There was also a significant effect of time*gender interaction on GAF scores, F(2.56,40.9) = 6.17, p=0.001, although there is no direct effect of gender (p=0.740). No direct effect of education or time*education interaction was found (p values >0.05). Additionally, baseline RAVLT-5 (r=0.725; p0.001), Stroop Time difference (r=-0.718; p0.001), WCST correct answers (r=0.644; r=0.003), category completed (r=0.630; p=0.004), and SANS scores (r=-0.427; p=0.42) significantly correlated with GAF in the 5th year. Among the CTQ subscores, physical abuse was significantly correlated with GAF in the 5th year (r=-0.415; p=0.009).

**Image 1:**

**Figure:**
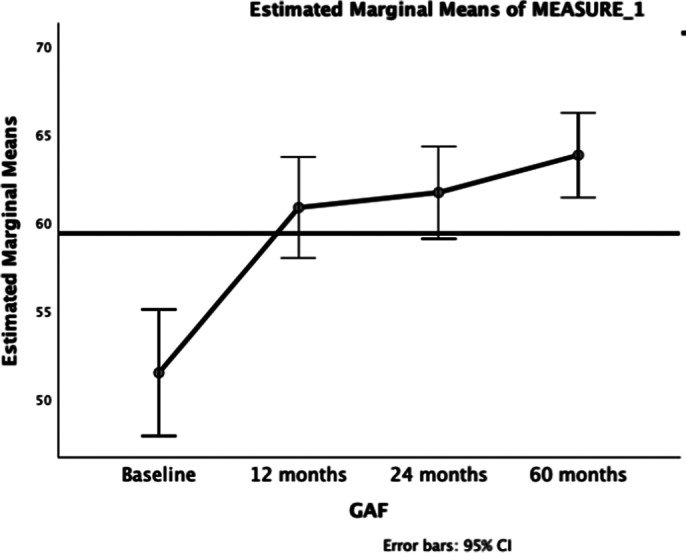

**Image 2:**

**Figure:**
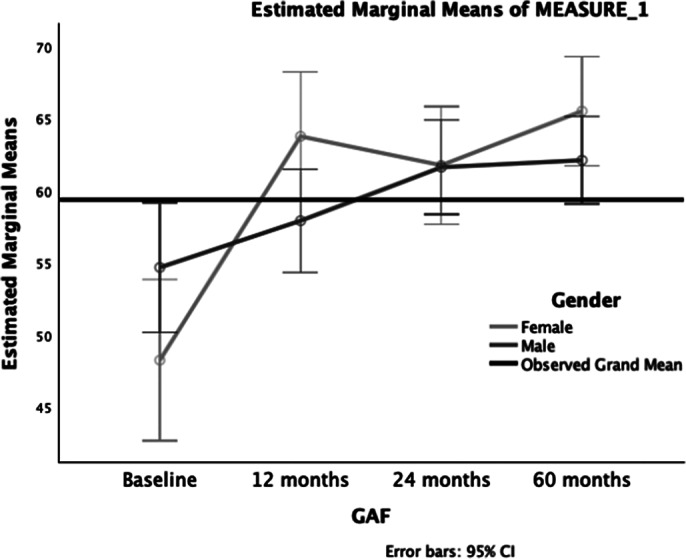

**Conclusions:**

GAF scores improved significantly over the 5-year follow-up in FES patients, with notable improvements occurring in the first year. Baseline cognitive performance, negative symptoms, and childhood trauma were found to be significant correlates of functioning, highlighting potential targets for early intervention.

**Disclosure of Interest:**

None Declared

